# Association between periodontal disease and hypertriglyceridemia: Propensity score matching analysis using the 7th Korea National Health and Nutrition Examination Survey

**DOI:** 10.1097/MD.0000000000036502

**Published:** 2023-12-22

**Authors:** Eun-Kyong Kim, Ju-Yeon Cho, Eun Young Park

**Affiliations:** a Department of Dental Hygiene, College of Science and Technology, Kyungpook National University, Sangju, South Korea; b Department of Dentistry, Dongsan Hospital, Keimyung University School of Medicine, Daegu, South Korea; c Department of Dentistry, Yeungnam University College of Medicine, Daegu, South Korea.

**Keywords:** hypertriglyceridemia, periodontitis, propensity score matching

## Abstract

The prevalence of periodontitis and dyslipidemia continues to increase, and several studies have reported an association between the 2. Therefore, we assessed the relationship between periodontitis and hypertriglyceridemia using propensity score matching to efficiently address confounding factors, as well as complex sample analysis with data from Korea National Health and Nutrition Examination Survey VII (2016–2018). To match the 1:1 ratio between the groups with and without periodontitis, the propensity scores of covariates, such as age, sex, education, income, smoking, drinking, obesity, and diabetes mellitus, were calculated using logistic regression. Both results of logistic regression analysis using complex sample design for whole and matched sample after propensity score matching demonstrated a significant association between hypertriglyceridemia and periodontitis, of which the adjusted odds ratio was 1.28 (95% confidence interval = 1.10–1.50) and 1.29 (95% confidence interval = 1.09–1.52), respectively. Our findings suggest that dental healthcare workers can help raise awareness among patients with periodontitis regarding the association between periodontitis and hypertriglyceridemia, which may help them manage the condition and receive treatment.

## 1. Introduction

Periodontal diseases are highly prevalent affecting 46% of adults aged 30 years or more in the United States^[1]^ and 26.8% of adults aged 19 years or more in Korea.^[2]^ Recently, the Health Insurance Review and Assessment Service announced that gingivitis and periodontal disease ranked first for 3 years in a row in the outpatient frequency of disease statistics. Periodontal disease is the most common and expensive outpatient disease.^[3]^ Moreover, periodontal diseases are chronic inflammatory diseases caused by dental plaque that leads to the destruction of the surrounding periodontal tissues and even tooth loss.^[4]^ Chronic diseases are ailments that are difficult to cure once they occur and must be managed for life. Biological, environmental, and socioeconomic factors often work in combination. In addition, overall treatment management is necessary as it relates bidirectionally to diseases with common factors. Therefore, identifying the relationship between periodontal and systemic diseases is essential for their effective and systematic control.^[5]^

Epidemiological studies of the relationship between periodontal and systemic diseases have been conducted since the 1980s.^[6]^ Numerous studies have demonstrated that periodontitis is associated with metabolic syndromes including obesity, diabetes, hypertension, and dyslipidemia.^[7–9]^ Dyslipidemia is a state of abnormal serum lipid profile, which results in elevated levels of total cholesterol, triglycerides (TG), low-density lipoproteins (LDL), and decreased levels of high-density lipoprotein (HDL) cholesterol.^[10]^

Periodontal disease and dyslipidemia are chronic inflammatory diseases with complex etiologies. Several epidemiological studies have reported an association between periodontitis and dyslipidemia. Moreover, recent studies have suggested a link between periodontal disease and dyslipidemia.^[11,12]^ In Korea, studies have been conducted to determine the relationship between dyslipidemia and periodontal disease, and it has been reported that hyperlipidemia and periodontal disease are closely associated.^[13–15]^ However, additional epidemiological studies are required to clarify the correlation between dyslipidemia and periodontitis. For example, large-scale data representative of a nation is necessary to assess the relationship between the aforementioned 2 chronic diseases. Therefore, some studies have attempted to evaluate this relationship using data from Korea National Health and Nutrition Examination Survey (KNHANES), a nationally representative survey. However, in such observational and cross-sectional studies, the reduction of bias due to confounding variables is important.

Propensity score matching (PSM) may help control for confounding variables. Furthermore, PSM reduces selective bias owing to the nonrandom assignment of exposure in an observational or prospective study, where it is difficult to apply a random assignment or adjust for confounding variables in cross-sectional studies to assess the relationship between exposure and outcome variables.^[16]^ The propensity score is defined as the probability of receiving a specific conditional exposure to the observed covariates. Matching according to propensity score may help adjust for covariate bias, resulting in an unbiased estimation of the effects of the independent variable.^[17,18]^ The most common implementation of PSM is a 1:1 match that forms pairs of case and control individuals.^[19]^ Therefore, this study examined the relationship between periodontal disease and hypertriglyceridemia using logistic regression analyses and 1:1 PSM with samples from the KNHANES to efficiently address confounding factors.

## 2. Methods

### 2.1. Survey and subjects

**Figure 1.**
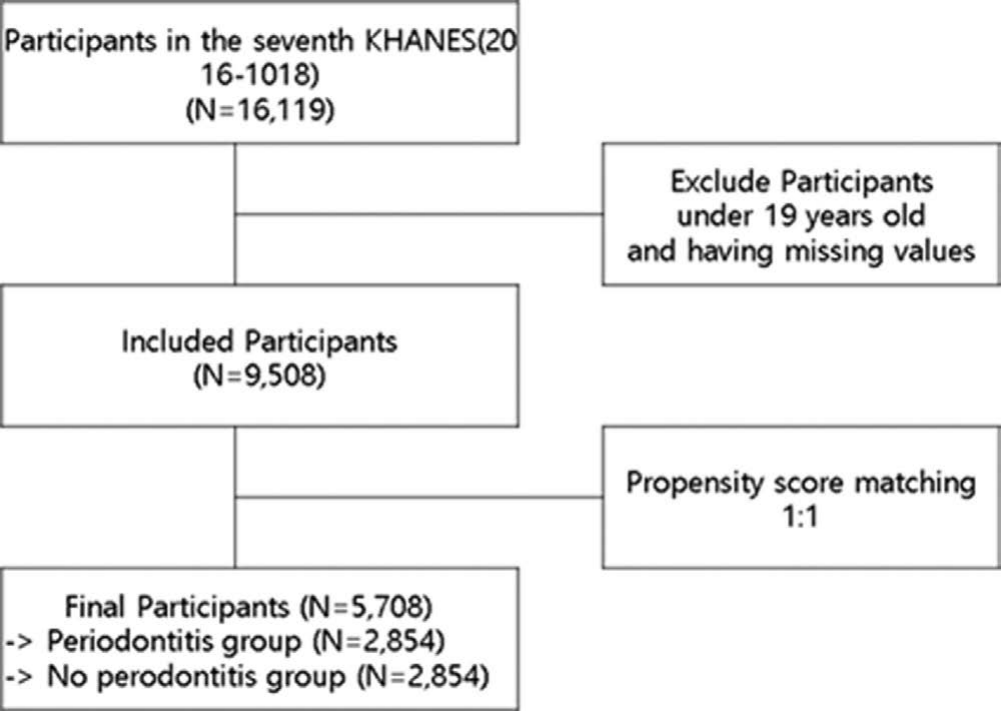
Flow of study.

Original data on the results of the KNHANES VII were downloaded from the official website of the KNHANES (https://knhanes.kdca.go.kr/knhanes/main.do) upon request. The 7^th^ KNHANES was conducted between 2016 and 2018 by the Korea Disease Control and Prevention Agency (KDCA), a nationally representative survey using stratified, complex, and multistage samples based on the Population and Housing Census.^[20]^ Sample weights were provided for the participants of KNHANES to represent whole population of Korea by KDCA. Demographic, social, and health information were surveyed using standardized questionnaires. In addition, clinical examinations were performed to assess general and oral health. Among the 16,119 individuals who participated in the survey, those aged < 19 years (n = 3290) and those with missing data (n = 3321) were excluded. Finally, data from 9508 individuals were analyzed using logistic analysis without PSM. Using PSM based on periodontitis, data from 5710 individuals were selected with 2855 participants per group (Fig. 1). The KNHANES VII protocol was approved by the Institutional Review Board of the KDCA (Institutional Review Board number: 2018-01-03-P-A). Written informed consent was obtained from all participants in this study. As the original data provided by the KDCA were secondary data without personally identifiable information, this study was exempt from approval by the Institutional Review Board of Kyungpook National University (KNU 2018-01-03-P-A). Only the dataset in anonymized form which were granted permission by the KDCA were provided and the study was performed in accordance with the Declaration of Helsinki.

### 2.2. Periodontal assessment

Specialized dentists trained according to the KNHANES protocol performed oral examinations to diagnose periodontitis using the Community Periodontal Index (CPI).^[21]^ The CPI scores were assessed for ten indexed teeth (numbers 17, 16, 11, 26, 27, 37, 36, 31, 46, and 47) using a CPI probe in a dental chair with light. Participants who had a CPI score of 3 or 4, (which meant periodontal tissue forming a periodontal pocket of ≥4 mm) among 10 indexed teeth were classified as periodontitis group. Others, with a CPI score of 0, 1, or 2, which indicated healthy gingiva, bleeding from gingiva, or calculus-formation in the periodontal tissue with a periodontal pocket < 4 mm, were classified as the healthy group.

### 2.3. Confounders

Sociodemographic characteristics such as age, sex, education level, and income, as well as behavioral characteristics of smoking and drinking, were surveyed using questionnaires by technical researchers. To adjust confounders, sociodemographic characteristics were classified as follows: age (19–49 or ≥50 years), gender (male or female), education level (elementary school, middle school, high school, or university), and income level (quartiles). In the case of smoking, those who had smoked more than 100 cigarettes in total during their life and were currently smoking were classified as yes, whereas others were classified as no. Drinking was categorized as yes for those who drank alcohol more than once per month over the past year. Individuals who consumed alcohol less than once per month during the previous year were classified as no.

### 2.4. Medical assessment

General diseases, such as obesity, hypertension, diabetes mellitus, hypercholesterolemia, and hypertriglyceridemia were evaluated using clinical examinations or laboratory procedures. In the case of general diseases, participants were categorized as either those who said yes or those who said no as per the guidelines provided by the KNHANES protocol. If the body mass index, calculated by dividing weight by height squared (kg/m^2^)^[22]^ was >25 kg/m^2^, obesity was defined as yes. In case participants (1) had a systolic blood pressure ≥140 mm Hg or diastolic blood pressure ≥90 mm Hg, respectively (2) were diagnosed with hypertension or received antihypertensive medication by a doctor, hypertension was marked as yes. In case participants (1) had a fasting blood glucose level ≥126 mg/dL, or (2) were diagnosed with diabetes mellitus or received anti-diabetic medication by a doctor, the diabetic was marked as yes. In case of hypercholesterolemia, participants with a total blood cholesterol level of ≥240 mg/dL or who received lipid-lowering medication from a doctor were categorized into the yes group. Finally, Hypertriglyceridemia was defined as yes if participants had a TG level ≥200 mg/dL or received triglyceride-lowering medication.

### 2.5. Statistical analyses

Complex sample analysis using whole participants as well as matched ones by PSM were performed to assess the association between periodontitis and hypertriglyceridemia. For complex sample analysis, we used sample weights based on stratification and clustering which were provided by KCDC according to KNHANES data profiles.^[20]^ We calculated the propensity scores of covariates such as age, sex, education, income, smoking, drinking, obesity, and diabetes mellitus using logistic regression for periodontitis.^[23]^ To match the 1:1 ratio, a greedy matching technique was applied with a caliper width of 0.1 without replacement. After PSM, the chi-square test and logistic regression adjusted for covariates such as age, sex, education, income, smoking, drinking, obesity, hypertension, and diabetes mellitus were done among matched participants using sample weights, stratification, and clustering which were provided by KCDC. All statistical analyses were performed using SAS (version 9.4; SAS Institute Inc., Cary, NC). As a *P* value, .05 was used which implies statistically significant.

## 3. Results

### 3.1. Prevalence of periodontal diseases according to covariates before and after PSM

Before PSM, the prevalence of periodontal diseases differed significantly according to age (*P* < .0001), sex (*P* < .0001), education (*P* < .0001), income (*P* < .0001), smoking (*P* < .0001), obesity (*P* < .0001), hypertension (*P* < .0001), diabetes (*P* < .0001), and hypertriglyceridemia (*P* < .0001; Table 1). After PSM, the prevalence of periodontal disease significantly differed according to sex (*P* = .006), smoking (*P* = .003), drinking (*P* = .034), diabetes (*P* = .008), and hypertriglyceridemia (*P* < .0001; Table 2).

**Table 1:** Characteristics of participants according to periodontitis before PSM (n = 9508).

Variables	Group	Periodontitis (N = 2982)		No periodontitis (N = 6526)		*P* value
		n	Weighted %	n	Weighted %	
Age	19 ≤ age ≤ 49	722	23.15	3766	57.16	<.0001
	50 ≤ age ≤ 59	2260	76.85	2760	42.84	
Sex	Male	1543	50.06	2479	36.74	<.0001
	Female	1439	49.94	4047	63.26	
Education	Elementary school	927	30.49	930	13.70	<.0001
	Middle school	409	14.41	506	7.80	
	High school	871	29.61	2173	33.98	
	University	775	25.49	2917	44.53	
Income	First quartile	778	25.98	984	14.73	<.0001
	Second quartile	810	26.82	1509	22.90	
	Third quartile	727	24.37	1958	29.71	
	Fourth quartile	667	22.83	2075	32.66	
Smoking	Current	2291	77.46	5641	86.77	<.0001
	None/ex-smoker	691	22.54	885	13.23	
Drinking	No	1387	46.97	3021	46.42	0.677
	Yes	1595	53.03	3505	53.58	
Obesity	No	1749	59.56	4496	69.65	<.0001
	Yes	1233	40.44	2030	30.35	
Hypertension	No	1640	55.54	4880	75.49	<.0001
	Yes	1342	44.46	1646	24.51	
Diabetes	No	2384	80.35	5963	91.89	<.0001
	Yes	598	19.65	563	8.11	
Hypertriglyceridemia	No	2407	80.62	5721	88.14	<.0001
	Yes	575	19.38	805	11.86	

PSM = propensity score matching.

*P* values are the results of the Chi-squared test using a complex sample design for whole sample.

**Table 2:** Characteristics of participants according to periodontitis after PSM (n = 5708).

Variables	Group	Periodontitis (N = 2855)		No periodontitis (N = 2855)		*P* value
		n	Weighted %	n	Weighted %	
Age	19 ≤ age ≤ 49	720	24.05	719	24.70	.63
	50 ≤ age ≤ 59	2134	75.95	2135	75.30	
Sex	Male	1446	48.94	1325	44.69	.006
	Female	1408	51.06	1529	55.31	
Education	Elementary school	866	29.83	824	28.00	.36
	Middle school	396	14.68	400	13.82	
	High school	834	29.53	876	31.90	
	University	758	25.96	754	26.28	
Income	First quartile	722	25.37	687	22.83	.27
	Second quartile	756	26.03	744	25.93	
	Third quartile	717	25.09	716	25.80	
	Fourth quartile	659	23.51	707	25.44	
Smoking	Current	2216	78.08	2326	82.08	.003
	None/ex-smoker	638	21.92	528	17.92	
Drinking	No	1350	47.58	1443	50.95	.034
	Yes	1504	52.42	1411	49.05	
Obesity	No	1698	60.57	1722	61.51	.538
	Yes	1156	39.43	1132	38.49	
Hypertension	No	1603	56.76	1606	57.65	.575
	Yes	1251	43.24	1248	42.35	
Diabetes	No	2308	81.28	2378	84.40	.008
	Yes	546	18.72	476	15.60	
Hypertriglyceridemia	No	2306	80.56	2406	85.03	.000
	Yes	548	19.44	448	14.97	

PSM = propensity score matching.

*P* values are the results of the Chi-squared test using a complex sample design for matched sample.

### 3.2. Associations between periodontitis and hypertriglyceridemia

The result of logistic regression analysis with a complex sample revealed that periodontitis was significantly associated with hypertriglyceridemia (odds ratio [OR] = 1.28; 95% confidence interval [CI] = 1.10–1.50) after adjusting for covariates including age, sex, education, income, smoking, drinking, obesity, hypertension, and diabetes. After PSM, a significant association between periodontitis and hypertriglyceridemia was still observed. The logistic regression analysis determined the association between periodontitis and hypertriglyceridemia among matched samples based on propensity scores derived from the model including age, sex, education, income, smoking, drinking, obesity, and hypercholesterolemia. Additionally, the same covariates were used in logistic regression analysis with a complex sample (OR = 1.29; 95 % CI = 1.09–1.52; Table 3)

**Table 3:** Logistic regression analyses for associations between periodontitis and hypertriglyceridemia.

Variables	Group	Before PSM (n = 9508)			After PSM (n = 5710)		
		OR*	95% CI		OR†	95% CI	
Periodontitis	Yes (ref = No)	1.28	1.10	1.50	1.29	1.09	1.52

All *P* values <.05.

CI = confidence interval, OR = odds ratio, PSM = propensity score matching.

* OR from logistic regression analysis with a complex sample design after adjusting for age, sex, education, income, smoking, drinking, obesity, hypertension, and diabetes mellitus for whole sample.

† OR from logistic regression analysis with a complex sample design after adjusting for age, sex, education, income, smoking, drinking, obesity, hypertension, and diabetes mellitus for matched sample.

## 4. Discussion and conclusions

In the present study, data derived from the 7^th^ KNHANES were used, and both complex sample analysis and PSM were conducted to evaluate the association between periodontitis and hypertriglyceridemia. The results of logistic regression analysis using complex sample analysis were used to detect a significant association between the 2 diseases (OR = 1.28, 95% CI = 1.10–1.50). The results of logistic regression analysis after PSM also revealed a significant association between periodontitis and hypertriglyceridemia (OR = 1.29, 95 % CI = 1.09–1.52). Based on the abovementioned results, we confirmed that periodontitis was associated with hypertriglyceridemia.

Dyslipidemia is an abnormality involving the plasma levels of TG, cholesterol, and lipoproteins. Moreover, dyslipidemia is typically observed in patients with obesity, metabolic syndrome, insulin resistance, and type 2 diabetes mellitus, and is known to be a risk marker for increased cardiovascular disease.^[24]^ Recently, an association between dyslipidemia and periodontal health has been reported. In a study of Japanese subjects, high triglyceride levels (>149 mg/dL) were a potential indicator of periodontal disease.^[25]^ Griffiths et al determined that high concentrations of LDL cholesterol and triglycerides were associated with periodontal disease.^[26]^ Conversely, periodontal disease is associated with a decrease in HDL cholesterol and an increase in LDL cholesterol, and periodontal inflammation impairs lipid metabolism.^[27]^

Inflammation may be a possible mechanism underlying the relationship between the 2 chronic diseases. Periodontitis is an inflammation of periodontal tissues caused by plaque microorganisms and metabolites, accompanied by systemic inflammation, and is presumed to affect systemic disease through 2 mechanisms. First, periodontal bacteria and their toxins directly circulate in the blood to cause systemic immune inflammation, and second, inflammatory mediators such as interleukin-1 (IL-1), IL-6, tumor necrosis factor-alpha (TNF-a) and C-reactive protein (CRP) are delivered to each tissue in the body through the bloodstream.^[28]^ Chronic inflammation is one of the most common causes of dyslipidemia. Chronic inflammation affects the dynamic balance among LDL, HDL, and TG levels, resulting in hypertriglyceridemia and dyslipidemia.^[27]^ Thus, periodontal disease and chronic inflammation may promote lipolysis and subsequent positive regulation of circulating triglycerides.^[29]^

Although the prevalence of dyslipidemia and periodontal disease is continuously increasing in Korea,^[30]^ few studies have investigated their association. Various confounding factors are reportedly associated with periodontitis and dyslipidemia,^[26]^ highlighting the need for large-scale well-controlled studies. In a previous domestic study, after controlling for factors such as age and obesity, the ratio of triglyceride to HDL cholesterol was positively correlated with the incidence of periodontal disease.^[31]^ In a logistic regression model, an increase in serum lipid triglyceride levels was reported to increase the risk of periodontitis.^[32]^ However, to apply the advantages of PSM to an unbiased estimation, we calculated the propensity score of important confounders concerning sociodemographic characteristics, health-related behaviors, and systemic diseases related to periodontitis and hypertriglyceridemia. After matching participants according to the calculated propensity score between the groups with and without periodontitis, we identified a significant correlation between periodontitis and hypertriglyceridemia.

However, this study had several limitations. First, it was a cross-sectional study. Second, we focused only on the relationship between periodontitis and hypertriglyceridemia. Therefore, a longitudinal study is necessary to investigate the effects of the duration of periodontitis on the incidence of hypertriglyceridemia. Third, PSM is limited by the quality of the propensity score model which depends on the selection, definition, and categorization of confounding predictors. Therefore, residual confounding factors may still be present and making true randomization impossible. Nevertheless, to our knowledge, this study is the first to qualitatively examine the association between periodontal disease and hypertriglyceridemia using PSM with large-scale KNHANES data, which represent national health data. Notably, we identified a significant association between hypertriglyceridemia and periodontitis. Furthermore, the results provide foundational evidence for future studies investigating the relationship between systemic and periodontal diseases. In conclusion, early awareness is important for patients with hypertriglyceridemia because it is a high-risk factor for coronary artery disease and atherosclerotic lesions.^[33]^ However, they are usually unaware of this and are discovered by chance during an examination. This chronic disease often has no symptoms and can be serious.^[34]^ Our findings suggest that dental healthcare workers can help raise awareness among patients with periodontitis regarding the association between periodontitis and hypertriglyceridemia, which may help them manage the disease or receive treatment.

## Author contributions

**Conceptualization:** Eun Young Park.

**Data curation:** Ju-Yeon Cho.

**Investigation:** Eun-Kyong Kim, Eun Young Park.

**Methodology:** Eun-Kyong Kim.

**Resources:** Ju-Yeon Cho.

**Software:** Eun-Kyong Kim.

**Supervision:** Eun-Kyong Kim, Ju-Yeon Cho.

**Visualization:** Eun-Kyong Kim.

**Validation:** Eun Young Park.

**Writing – original draft:** Eun Young Park.

**Writing – review & editing:** Eun-Kyong Kim, Ju-Yeon Cho, Eun Young Park
